# Impact of Positive Airway Pressure and Mask Leakage on Dry Eye and Glaucoma Risk in Obstructive Sleep Apnea: A Cross-Sectional Analysis

**DOI:** 10.3390/biomedicines13123077

**Published:** 2025-12-13

**Authors:** Wei-Xiang Wang, Ya-Ning Chuang, Chen-Ni Chang, Mei-Chen Yang, Elizabeth P. Shen

**Affiliations:** 1 Department of Education, Taipei Tzu Chi Hospital, Buddhist Tzu Chi Medical Foundation, New Taipei City 231, Taiwan; b101106012@tmu.edu.tw; 2 Department of Ophthalmology, Taipei Tzu Chi Hospital, Buddhist Tzu Chi Medical Foundation, New Taipei City 231, Taiwan; annie721176@gmail.com; 3 Department of Emergency Medicine, Taipei Tzu Chi Hospital, Buddhist Tzu Chi Medical Foundation, New Taipei City 231, Taiwan; chenni6060@gmail.com; 4 School of Medicine, Tzu Chi University, Hua-Lien City 970, Taiwan; mimimai3461@gmail.com; 5 Division of Pulmonary Medicine, Department of Internal Medicine, Taipei Tzu Chi Hospital, Buddhist Tzu Chi Medical Foundation, New Taipei City 231, Taiwan; 6 Department of Ophthalmology, National Taiwan University Hospital, College of Medicine, National Taiwan University, Taipei City 100, Taiwan

**Keywords:** dry eye disease, glaucoma, continuous positive airway pressure, obstructive sleep apnea, PAP leakage, air leak

## Abstract

**Purpose:** This study investigates the association between obstructive sleep apnea (OSA), dry eye disease (DED), and glaucoma, focusing on the impact of positive airway pressure (PAP) usage and air leakage. **Methods:** This retrospective cross-sectional study included 57 adults with polysomnography-confirmed OSA between 2010 and 2023. Participants were grouped into PAP users (PAP+, *n* = 40) and non-users (PAP−, *n* = 17). Ocular assessments included tear film break-up time, Schirmer’s test, Oxford staining, meibomian gland evaluation, intraocular pressure, cup-to-disc (C/D) ratio, and retinal nerve fiber layer thickness. PAP device data (usage duration and air leak rate) and OSA severity metrics were recorded. Group comparisons used chi-square and Student’s *t*-test, and regression analyses identified associations between PAP leakage and ocular parameters. **Results:** Among the 57 OSA patients, PAP users showed a trend toward a higher risk of glaucoma (OR = 0.83) and DED (OR = 0.69) compared to non-users, but neither trend was statistically significant. PAP users had significantly more severe OSA, including longer N1 sleep stage (*p* = 0.0005), higher apnea-hypopnea index (AHI, *p* = 0.0001), and poorer oxygenation. PAP leakage: 95% (mean = 25.84 L/min) exceeded the 24 L/min threshold specified in ResMed’s clinical guidelines, suggesting suboptimal therapy. Higher PAP leak was significantly associated with a lower Schirmer’s test value (*p* = 0.031) and a higher C/D ratio (*p* = 0.040) on regression analysis. However, no significant differences were found in ophthalmic parameters between PAP+ and PAP− groups. **Conclusions:** Suboptimal PAP therapy as mask leakage or nocturnal hemodynamic changes may worsen evaporative dry eye and affect intraocular pressure. Our findings highlight the association between PAP mask leakage and reduced tear production, and suggest that OSA-related optic nerve stress may persist unless both hypoxia and nocturnal IOP fluctuations are properly managed. However, due to the relatively small sample size and retrospective cross-sectional design, future prospective studies with larger cohorts are needed to confirm these associations.

## 1. Introduction

Obstructive sleep apnea (OSA) is a prevalent sleep disorder marked by recurrent episodes of apnea and hypopnea that cause arousals and oxygen desaturation due to transient upper airway collapse. In the long term, OSA leads to intermittent hypoxia, oxidative stress, and systemic inflammation [1,2]. These physiological disturbances have been linked to various systemic and ocular diseases [3,4].

Dry eye disease (DED) is commonly observed in OSA patients, possibly due to autonomic dysregulation and ocular surface inflammation [5,6]. Additionally, positive airway pressure (PAP) therapy as the first-line treatment for moderate to severe OSA can inadvertently direct airflow toward the eyes if the mask does not seal properly [7]. This airflow may disrupt the tear film and ocular surface. Indeed, up to 50% of continuous positive airway pressure (CPAP) users report eye irritation, and around 20% specifically attribute it to mask air leaks [8,9].

Glaucoma, an irreversible optic neuropathy, has also been linked to OSA. Intermittent hypoxemia in OSA is thought to induce oxidative stress and impair ocular perfusion, contributing to glaucomatous optic nerve damage [10]. Research indicates that individuals with OSA are 40% more likely to develop glaucoma compared to those without OSA [11]. The impact of PAP therapy on glaucoma progression remains controversial. Some studies suggest that PAP use may increase intraocular pressure (IOP) and accelerate glaucoma progression. However, other research indicates that PAP improves oxygenation and reduces systemic inflammation, potentially slowing glaucoma progression [12,13,14].

This study primarily aimed to assess the association between PAP mask leakage and relevant ophthalmic parameters, and secondarily to compare dry eye and glaucoma indicators between OSA patients with and without PAP therapy.

## 2. Materials and Methods

### 2.1. Study Design

This retrospective cross-sectional study was conducted at Taipei Tzu Chi Hospital, Buddhist Tzu Chi Medical Foundation, New Taipei, Taiwan, using real-world data from the hospital’s database between 2010 and 2023. Approval was granted by the Institutional Review Board of Taipei Tzu Chi Hospital (IRB: 11-XD-051). The approved date is 11 July 2022.

### 2.2. Inclusion and Exclusion Criteria

All participants were adults (≥20 years) with obstructive sleep apnea confirmed by overnight polysomnography (PSG). For inclusion, patients in the PAP+ group were required to have received positive airway pressure therapy between 2010 and August 2023 for at least one year with good adherence (defined as ≥4 h of use per night on ≥70% of nights), to ensure adequate treatment exposure. For PAP+ patients, a follow-up sleep study was performed after at least one year of PAP therapy. The latest PSG for these patients was performed between March 2022 and August 2023, and all PAP users underwent a comprehensive ophthalmologic evaluation within three months after the PSG examination. For the OSA without PAP group, participants were patients with OSA who underwent comprehensive ophthalmologic examinations between March 2022 and August 2023 but had never received PAP therapy. Exclusion criteria included patients lacking essential ophthalmic data or having any history of refractive surgery (such as laser-assisted in situ keratomileusis or small incision lenticle extraction) that could affect ocular surface measurements [15,16].

### 2.3. Patient Selection and Grouping

A total of 57 OSA patients met the inclusion criteria. Of these, 40 patients had used PAP therapy (PAP+ group) and 17 had not used PAP (PAP− group). All PAP+ patients had been on automatic positive airway pressure (APAP) or CPAP therapy for ≥1 year. We collected data on comorbid conditions and personal history, including hypertension, diabetes mellitus (DM), dyslipidemia, coronary artery disease (CAD), cerebrovascular accident (CVA), chronic obstruction pulmonary disease (COPD), asthma, chronic kidney disease (CKD), dialysis, alcohol consumption, cigarette use, occurrence of glaucoma, and DED.

### 2.4. Data Collection

PSG was conducted within our hospital’s sleep center. All PAP devices were manufactured by ResMed (San Diego, CA, USA) and included both APAP (AirSense 10 AutoSet and S9 AutoSet) and CPAP (AirSense 10 CPAP and S9 Elite). In accordance with ResMed clinical guidelines, large leak was defined as 95th-percentile unintentional leak > 24 L/min. Although mask type was not uniformly documented, all PAP devices reported the 95th-percentile unintentional leak (L/min), which is standardized across PAP and mask types. Therefore, this parameter was considered comparable among subjects. During the procedure, we recorded various sleep parameters, including rapid eye movement (REM) sleep, nasal and oral airflow, mean arterial oxygen saturation (Mean SaO_2_), nadir SaO_2_, cumulative time below 90% oxygen saturation (CT90), and autonomic nervous system activity (measured by low and high-frequency power). OSA severity was assessed by the apnea-hypopnea index (AHI) as mild (5–14.9), moderate (15–29.9), or severe (≥30).

At the initial referral visit, participants underwent a comprehensive ophthalmic evaluation, including best-corrected visual acuity (BCVA) to assess visual function (clinically significantly worse if more than 0.3 LogMAR), intraocular pressure (IOP) measurement for glaucoma screening (abnormal if >21 mmHg), tear break-up time (TBUT) to evaluate tear film stability (dry eye if <5 s), and the Oxford staining score to grade ocular surface damage (clinically significant if ≥ grade 2). Conjunctival and lid vascularity, plugging of Meibomian gland orifices, lid margin irregularity, and lid thickening were examined as indicators of meibomian gland dysfunction. Schirmer’s test was performed to measure tear production (abnormal if ≤10 mm/5 min), and the Ocular Surface Disease Index (OSDI) questionnaire was used to quantify dry eye symptoms (clinically significant if >13 points). To assess structural changes related to glaucoma, the cup-to-disc (C/D) ratio (abnormal if ≥0.5 or asymmetry > 0.2), central retinal thickness (CRT) (reduction < 240 µm as clinical significant), and retinal nerve fiber layer (RNFL) thickness (reduction < 85 µm suggestive of early glaucomatous change) were measured using optical coherence tomography (OCT). These parameters were used to evaluate the impact of PAP usage and mask leakage on ocular surface and optic nerve function.

### 2.5. Statistical Analyses

All statistical analyses were performed using MedCalc version 23.0.1 (MedCalc Software Ltd., Ostend, Belgium). Chi-square tests were used to analyze categorical variables, Student’s *t*-tests were used for continuous variables with normal distribution, and Mann–Whitney U tests were applied for continuous variables that were not normally distributed. Categorical data are reported as counts and percentages, and continuous data as mean ± standard deviation (SD). Logistic regression was used to estimate odds ratios (ORs) and 95% confidence intervals for the presence of DED and glaucoma. ANOVA tests were conducted to compare OSA severity (AHI groups) and PAP-use group with ocular parameters, including OSDI, Oxford staining, TBUT, Schirmer’s test, C/D ratio, and RNFL thickness. A two-tailed *p*-value < 0.05 was considered statistically significant.

The primary objective focused on the association between the degree of PAP mask leakage and relevant parameters, and the secondary exploratory objective was the comparison of ophthalmic parameters between the PAP+ and PAP− groups.

## 3. Results

### 3.1. Demographic Characteristics

The study included 57 OSA patients, with 40 patients receiving PAP therapy (PAP+) and 17 patients not receiving PAP therapy (PAP−). The PAP+ group had a significantly higher proportion of males than the PAP− group (80% vs. 52.9%, *p* = 0.042) (Table 1). The mean age of participants was similar between groups (*p* = 0.586). Regarding comorbidities, the prevalence of hypertension, DM, dyslipidemia, CAD, CVA, asthma, CKD, dialysis, alcohol consumption, cigarette use, glaucoma, and DED showed no significant difference between groups. However, COPD was significantly more common in the PAP+ group (55% vs. 23.5%, *p* = 0.029) (Table 1).

The OR for glaucoma (OR = 0.83, 95%CI 0.27–2.60) and DED (OR = 0.69, 95%CI 0.17–2.76) indicated a trend where PAP+ group may have a higher risk, though the differences were not statistically significant (Table 2). PAP use duration was 6.8 ± 3.5 years (range 1–14 years), and the average PAP usage each day was 6.0 ± 1.4 h per night (Table 2). Among 57 patients, the majority (68.4%) had severe OSA, 14.0% had moderate OSA, and 17.5% had mild OSA (Table 3).

### 3.2. Sleep Study Findings

PSG data revealed that the PAP+ group had more severe sleep-disordered breathing and poorer sleep quality than the PAP− group (Table 4). The PAP+ group had a significantly higher percentage of sleep in stage N1 (33.4%, *p* = 0.0005) but showed no significant differences in other sleep stages (Table 4). They also exhibited a much higher residual AHI (*p* = 0.0001), indicating more frequent sleep disruptions. Oxygenation was markedly worse in the PAP+ group, with lower mean SaO_2_ (92.03%), lower nadir SaO_2_ (76.15%), and higher CT90 (23.64%), indicating poorer oxygenation during sleep (Table 4). Sympathetic and parasympathetic activity were similar between the groups.

**Figure 1:**
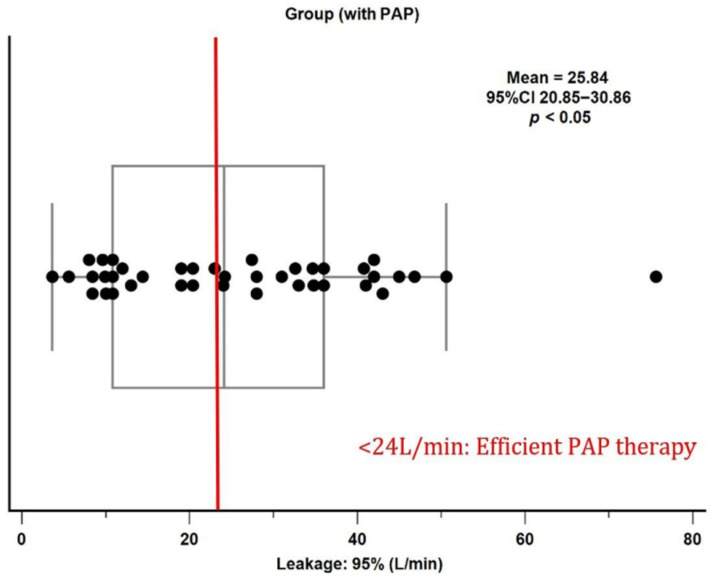
PAP leakage: 95% (L/min) of the PAP+ group (black dots). The red vertical line marks the 24 L/min threshold, indicating the cutoff for efficient PAP therapy.

Among the PAP+ group, mask leak was a notable issue. The average 95th-percentile unintentional leak was 25.84 L/min (95% CI 20.85–30.86), exceeding the recommended threshold of 24 L/min (Figure 1). This finding suggests that many PAP users had suboptimal mask fit or seal, potentially compromising therapy efficacy and comfort.

### 3.3. Association Between PAP Leakage and Relevant Parameters

In the regression analysis, the Schirmer’s test (coefficient = −1.33, *p* = 0.0307) and C/D ratio (coefficient = 0.41, *p* = 0.0402) showed statistically significant associations, indicating reduced tear production and greater optic disc cupping with higher leak (Table 5). Other outcomes, including low-frequency power (%), nadir SaO_2_ (%), CT90 (%), OSDI, Oxford staining, TBUT, RNFL thickness, and CRT, did not show significant linear relationship with leak (all *p* > 0.05) (Table 5). In addition, an expected negative correlation was observed between oxygen saturation metrics: CT90 (%) and nadir SaO_2_ (%) (Pearson *r* = −0.32, *p* = 0.043; Supplementary Figure S1).

### 3.4. Ophthalmic Findings Between PAP+ and PAP− Groups

Baseline ophthalmic evaluations showed no significant differences between PAP+ and PAP− groups (Table 6). Best-corrected visual acuity (Log MAR), IOP, tear film stability (TBUT, Schirmer test), ocular surface parameters (Oxford staining), dry eye symptom questionnaire (OSDI), eyelid-related parameters (lid vascularity, irregularity, thickening, and gland orifice plugging), and structural indices (C/D ratio, CRT, and RNFL thickness) were similar between groups (all *p* > 0.05). These findings suggest that, in a real-world setting where variables and prescribed treatments are not controlled, the overall outcomes showed no significant difference among patients.

### 3.5. Subgroup Analysis: ANOVA

The ANOVA subgroup analysis compared ocular surface and structural parameters across PAP users and different OSA severity groups (mild, moderate, severe) (Supplementary Figure S2). No significant differences were observed in OSDI, Oxford staining, TBUT, Schirmer test, C/D ratio, or RNFL thickness among the groups. However, the figure showed that the overall distribution of the PAP+ group was similar to that of the severe OSA group (Supplementary Figure S2).

## 4. Discussion

In this study, increasing PAP mask leakage showed significant association with ocular changes: lower Schirmer’s test scores and a higher C/D ratio, indicating reduced tear production and greater optic nerve cupping. In contrast, we did not find significant differences between PAP users and non-users in the risk of DED or glaucoma, nor in most ocular measurements. One explanation is that the PAP+ group had inherently more severe OSA and thus a higher ocular risk profile at baseline, which could have masked any protective or adverse effects of PAP itself. Indeed, PAP+ group had worse sleep parameters than PAP− group, with higher AHI and more severe hypoxemia. Moreover, many users also had mask leakage exceeding 24 L/min, suggesting suboptimal OSA control despite long-term PAP therapy. Additionally, ANOVA revealed the ocular outcomes in the PAP+ group were similar to the severe OSA group, reinforcing OSA severity as an important factor. Therefore, while PAP therapy did not show a clear net benefit or harm to ocular health in this real-world setting, the presence of high mask leak and severe OSA were associated with signs of evaporative dry eye and possible glaucomatous changes.

### 4.1. PAP Therapy and Dry Eye Disease

OSA is known to provoke systemic inflammation and autonomic imbalances that can adversely affect the ocular surface [17]. Elevated levels of inflammatory cytokines such as IL-6 and TNF-α have been documented in OSA patients [18], and these inflammatory mediators have also been found in higher concentrations on the ocular surface of OSA patients, contributing to tear film instability and reduced tear production [4,19]. Treatment with PAP can improve oxygenation and reduce systemic inflammation, which in theory should benefit dry eye. However, if pressurized air from a PAP mask leaks into the eyes, it can cause excessive evaporation of the tear film, leading to tear hyperosmolarity and ocular surface irritation and inflammation [20,21]. In our study, TBUT did not differ significantly between PAP users and non-users, likely because tear breakup was measured during daytime clinic visits rather than immediately after PAP use. Compared with TBUT, the Schirmer test reflects overall tear secretion and is less influenced by short-term environmental factors or blinking patterns; therefore, it may serve as a more reliable indicator of sustained tear deficiency several hours after PAP use [22]. The reduced Schirmer values associated with higher leak rates suggest that repeated nightly exposure to airflow can induce chronic ocular surface inflammation and diminished lacrimal gland output [23].

Recent studies support our findings on PAP-related dry eye. Fu et al. reported that Taiwanese OSA patients using CPAP had an almost fourfold higher risk of developing DED compared to those without CPAP [21]. Likewise, a large real-world study based on U.S. insurance data showed that OSA patients using CPAP or other mask devices had a higher incidence of dry eye compared to the general population, with a prevalence of 6% after one year and 13% after three years [24]. Another study by Hayirci et al. demonstrated that after just four months of CPAP treatment, patients showed increased corneal staining, conjunctival squamous metaplasia, and higher tear evaporation rates, all consistent with an aggravation of evaporative dry eye from the CPAP airflow [23].

### 4.2. PAP Therapy and Glaucoma

OSA has been identified as a risk factor for glaucoma through intermittent hypoxia and fluctuating perfusion to the optic nerve [13]. During apnea episodes, decreased oxygenation and increased sympathetic nervous system activity cause sharp fluctuations in blood pressure and intracranial pressure, leading to long-term retinal ganglion cell damage [11,25]. Effective PAP therapy can prevent apneic events and thereby should mitigate these oscillations [26,27,28]. On the other hand, the nighttime PAP use might impede venous outflow from the orbit or raise episcleral venous pressure, potentially increasing IOP during the night [12,29]. Our finding that higher PAP leak was associated with larger C/D ratio raises the possibility that patients with poor PAP adherence or effectiveness may experience ongoing optic nerve stress. Residual OSA events in the setting of ineffective PAP could lead to persistent nocturnal hypoxia and blood pressure surges, counteracting any protective effect of PAP on the eyes.

Large-scale data have not found a definitive reduction in glaucoma incidence with PAP therapy. A large cohort study found that CPAP therapy did not significantly reduce glaucoma incidence compared to the control group [12]. Similarly, Kiekens et al. reported that while CPAP improves breathing, it may also lead to increased nocturnal IOP and reduced ocular perfusion pressure during sleep [30]. Consistent with these observations, our results showed no clear protective effect of PAP on glaucoma metrics, as evidenced by a similar rate of glaucoma between the PAP− and PAP+ groups (OR = 0.83). Overall, while untreated OSA is a known risk factor for glaucomatous damage, PAP therapy alone may not fully eliminate this risk, especially if issues like mask leakage and pressure-related IOP changes are not addressed.

### 4.3. Limitations

Our study has several limitations. First, this cohort included all eligible patients from a single center; however, the relatively small sample size and uneven gender distribution may limit the generalizability of the findings. Nevertheless, these findings may represent a real-world scenario of patients using PAP for 6.8 years (range 1–14 years). Second, the retrospective cross-sectional design makes it difficult to establish causality or observe longitudinal changes resulting from PAP therapy. Therefore, the effects of PAP on ocular parameters or sleep-related symptoms may not be directly inferred. Third, this real-world observational study increases the generalizability of the findings, but it also means that variables were not controlled; consequently, the PAP user group had more severe OSA on average and similar outcomes to the severe OSA group in ANOVA analysis, reflecting clinical practice. Despite these limitations, our study is, to our knowledge, the first to quantitatively link objective PAP leak measurements with ocular surface and optic nerve parameters in OSA. The insights highlight the importance of considering mask fit and air leakage in future research and in the clinical management of OSA patients to protect ocular health. Future prospective, multicenter studies with larger cohorts and standardized follow-up are warranted to confirm the associations observed here and to derive generalizable conclusions.

## 5. Conclusions

In summary, effective PAP therapy alleviates hypoxia and systemic stress in OSA patients, which should theoretically benefit the eyes. However, if PAP treatment is suboptimal, particularly in the presence of mask leakage or persistent nocturnal oxygen desaturation, it may exacerbate evaporative dry eye and could contribute to glaucoma-related stress on the optic nerve. Our findings emphasize the association between PAP mask leakage and ocular surface dryness, along with lower Schirmer’s test scores indicating reduced tear production, and suggest that OSA-related optic neuropathy may progress due to both hypoxemia and nocturnal IOP fluctuations. Optimizing PAP device fit and adherence is not only crucial for controlling sleep apnea but also important for safeguarding long-term ocular health. However, due to the relatively small sample size and retrospective cross-sectional design, future prospective studies with larger cohorts are needed to confirm these associations.

## Figures and Tables

**Table 1:** Clinical characteristics of the study participants.

	PAP (−), *n* = 17	PAP (+), *n* = 40	*p*-Value
Women, *n* (%)	8(47.1)	8(20)	0.042 *
Men, *n* (%)	9(52.9)	32(80)	0.042 *
Age(year)(mean ± SD)	65.12 ± 10.42	65.35 ± 11.13	0.586
Hypertension, *n* (%)	10(58.5)	26(65)	0.658
Diabetes mellitus, *n* (%)	5(29.4)	10(25)	0.484
Dyslipidemia, *n* (%)	6(35.3)	9(22.5)	0.247
Coronary artery disease, *n* (%)	1(5.9)	11(27.5)	0.063
Cerebrovascular accident, *n* (%)	1(5.9)	5(12.5)	0.414
COPD, *n* (%)	4(23.5)	22(55)	0.029*
Asthma, *n* (%)	1(5.9)	15(40)	0.317
Chronic kidney disease, *n* (%)	2(11.8)	4(10)	0.586
Dialysis, *n* (%)	1(5.9)	0(0)	0.298
Glaucoma, *n* (%)	4(23.5)	7(17.9)	0.401
Dry eye disease, *n* (%)	8(47)	17(42.5)	0.132
Alcohol, *n* (%)	3(17.6)	9(22.5)	0.489
Cigarette, *n* (%)	6(35.3)	20(51.3)	0.270

* means statistical significance; PAP = positive air pressure; COPD = chronic obstructive pulmonary disease.

**Table 2:** The odds ratio for glaucoma and DED, and duration of PAP use.

	PAP (−), *n* = 17	PAP (+), *n* = 40	Odds Ratio, 95%Cl
Glaucoma, *n* (%)	4 (23.5)	7 (17.9)	0.83 (0.27, 2.60)
Dry eye disease, *n* (%)	8 (47)	17 (42.5)	0.69 (0.17, 2.76)
PAP use duration, years (Min–Max)	0	6.8 ± 3.5 (1–14)	

Cl = confidence interval; Min = minimal; Max = maximal.

**Table 3:** The distribution of OSA severity and its association with DED and glaucoma.

OSA Severity	AHI	OSA Patients Among Different Severities (*n*)	DED Patients Among Different Severities (*n*, %)	Glaucoma Patients Among Different Severities (*n*, %)
Normal	0—5	0	0	0
Mild	5—15	10	7 (70%)	2 (20%)
Moderate	15—30	8	3 (37.5%)	4 (50%)
Severe	≥30	39	15 (38.5%)	5 (12.8%)

AHI = apnea-hypopnea index; OSA = obstructive sleep apnea; DED = dry eye disease

**Table 4:** Polysomnography data of the PAP+ and PAP− Groups.

Polysomnography Data		PAP (−)			PAP (+)			
		*n*	Mean	±SD	*n*	Mean	±SD	*p*-Value
Sleep quality	Sleep stage N1 (%)	17	15.98	±7.98	40	33.40	±26.89	0.0005 *
	Sleep stage N2 (%)	17	60.30	±12.26	40	55.14	±13.62	0.184
	Sleep stage N3 (%)	17	7.65	±9.35	40	3.20	±5.22	0.0799
	REM sleep (%)	17	16.10	±9.47	40	11.93	±8.34	0.1025
OSA severity	Residual AHI (event/hour)	17	24.85	±14.17	40	46.54	±23.96	0.0001 *
Oxygenation	Mean SaO_2_ (%)	17	93.76	±1.79	40	92.03	±3.14	0.0111 *
	Nadir SaO_2_ (%)	17	83.06	±5.36	40	76.15	±7.05	0.0007 *
	CT90 (%)	17	6.99	±10.75	40	23.64	±22.83	0.0005 *
Sympathetic activity	Low frequency power (%)	17	53.64	±8.89	40	58.14	±13.63	0.2192
Parasympathetic activity	High frequency power (%)	17	46.36	±8.89	40	41.86	±13.63	0.2192

* means statistical significance; REM = rapid eye movement; AHI = apnea-hypopnea index; SaO_2_ = oxygen saturation; CT90 = cumulative time below 90% oxygen saturation.

**Table 5:** The regression analysis examined the relationship between PAP leakage: 95% and marked PSG parameters and ophthalmic outcomes.

	PAP Leakage: 95%		
Outcomes	Coefficient	95%Cl	*p*-Value
Low-frequency power (%)	−0.15	−0.63–0.32	0.5103
Nadir SaO_2_ (%)	0.02	−0.98–1.02	0.9654
CT90 (%)	0.12	−0.17–0.40	0.4031
OSDI	−0.04	−0.38–0.29	0.8030
Oxford staining	−2.04	−9.77–5.70	0.5916
TBUT	0.29	−4.53–5.12	0.9007
Schirmer test (mm)	−1.33	−2.53–−0.14	0.0307 *
C/D ratio	0.41	0.02–0.79	0.0402 *
CRT (μm)	0.11	−0.15–0.37	0.4009
RNFL thickness (μm)	0.24	−0.39–0.89	0.4400

* means statistical significance; CT90 = cumulative time below 90% oxygen saturation; OSDI = Ocular Surface Disease Index; TBUT = tear break-up time; C/D ratio = cup-to-disc ratio; CRT = central retinal thickness; RNFL = retinal nerve fiber layer.

**Table 6:** Comparison of ophthalmic parameters between PAP+ and PAP− groups.

	PAP(−)			PAP(+)			
Ophthalmic Outcomes	*n*	Mean	±SD	*n*	Mean	±SD	*p*-Value
LogMAR	17	0.25	±0.23	40	0.24	±0.15	0.9287
IOP (mmHg)	17	15.00	±3.66	40	15.39	±3.19	0.6902
TUBT	17	3.58	±1.85	40	3.78	±1.71	0.6991
Oxford staining	17	0.42	±0.69	40	0.57	±0.96	0.5434
Vascularity	17	1.90	±0.96	40	1.52	±1.01	0.1960
Plugging of gland orifice	17	1.74	±0.73	40	1.61	±0.87	0.6110
Irregularity	17	0.44	±0.50	40	0.39	±0.66	0.7663
Thickening	17	0.24	±0.44	40	0.23	±0.44	0.9355
Shirmer test (mm)	17	8.26	±5.07	40	7.84	±5.77	0.7921
OSDI	17	20.32	±17.61	40	16.59	±20.54	0.5165
C/D ratio	17	0.56	±0.13	40	0.49	±0.18	0.1750
CRT (μm)	17	250.77	±25.07	40	256.00	±29.67	0.5519
RNFL thickness (μm)	17	89.83	±12.40	40	96.28	±10.57	0.0627

LogMAR = logarithm of minimum angle of resolution; IOP = intraocular pressure; TBUT = tear break-up time; OSDI = Ocular Surface Disease Index; C/D ratio = cup-to-disc ratio; CRT = central retinal thickness; RNFL = retinal nerve fiber layer.

## Data Availability

The data presented in this study are available on request from the corresponding authors.

## Supplementary Materials

The following supporting information can be downloaded at: https://www.mdpi.com/article/10.3390/biomedicines13123077/s1, Figure S1: Negative correlation between oxygen saturation metrics: CT90 (%) and nadir SaO_2_ (%); Figure S2: The ANOVA subgroup analysis compared ocular surface and structural parameters across PAP users and different OSA severity groups (mild, moderate, severe).

## Abbreviations

The following abbreviations are used in this manuscript:

AHI	apnea-hypopnea index
APAP	automatic positive airway pressure
BCVA	best-corrected visual acuity
C/D	cup-to-disc ratio
CAD	coronary artery disease
CKD	chronic kidney disease
COPD	chronic obstruction pulmonary disease
CPAP	continuous positive airway pressure
CRT	central retinal thickness
CVA	cerebrovascular accident
CT90	cumulative time below 90% oxygen saturation
DED	dry eye disease
DM	diabetes mellitus
IOP	intraocular pressure
SaO_2_	Oxygen saturation
OCT	optical coherence tomography
OR	odds ratio
OSDI	Ocular Surface Disease Index
OSA	obstructive sleep apnea
PAP	positive airway pressure
PSG	polysomnography
REM	rapid eye movement
RNFL	retinal nerve fiber layer thickness
SD	standard deviation
TBUT	tear break-up time
