# Supplementary figures and images for: Impact of Positive Airway Pressure and Mask Leakage on Dry Eye and Glaucoma Risk in Obstructive Sleep Apnea: A Cross-Sectional Analysis
Source: Biomedicines. 2025 Dec 13;13(12):3077. doi: 10.3390/biomedicines13123077 (PMC12731221; doi:10.3390/biomedicines13123077)

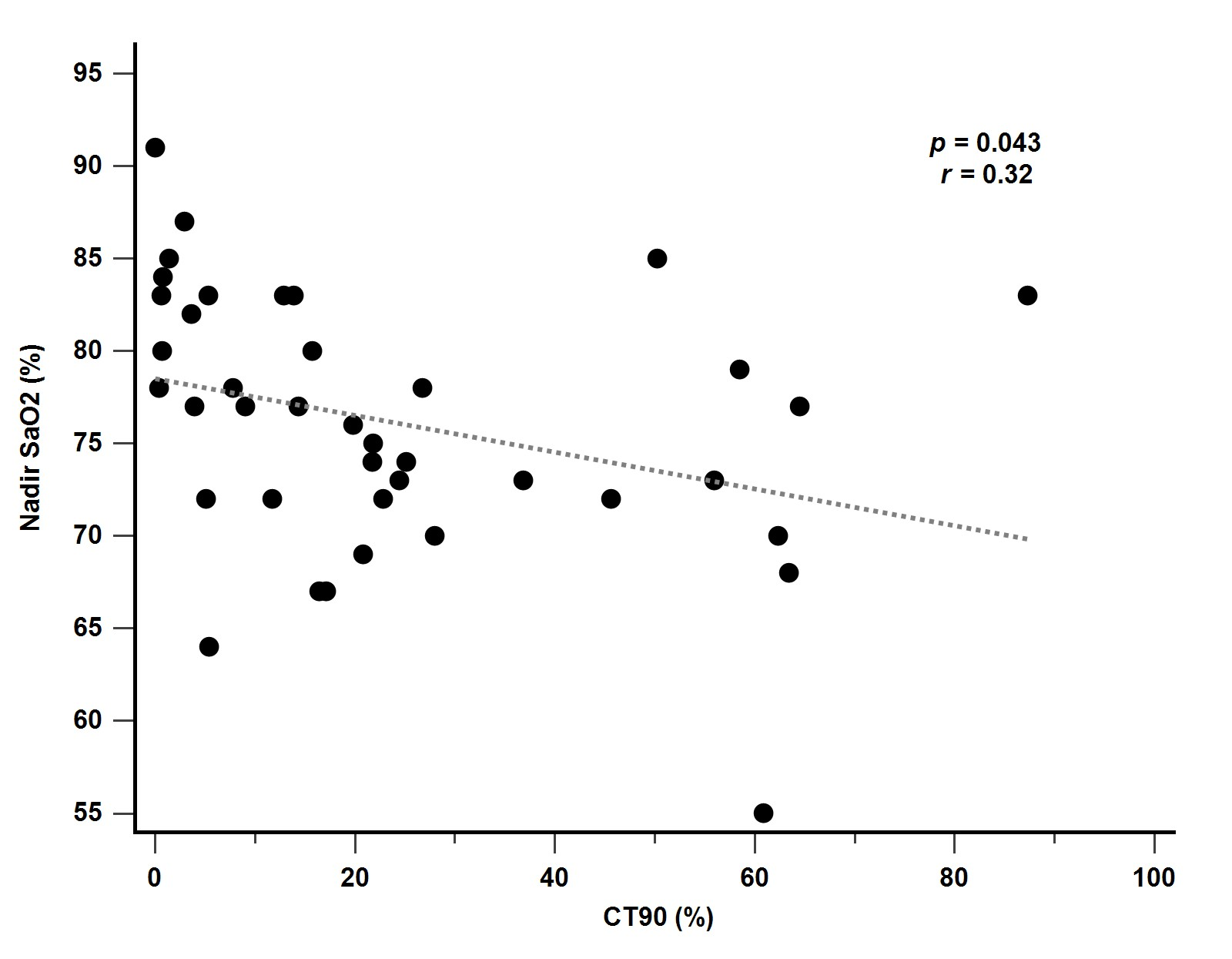

Supplement: Supplementary file 1 [file biomedicines-13-03077-s001.zip › Figure. S1.tif]

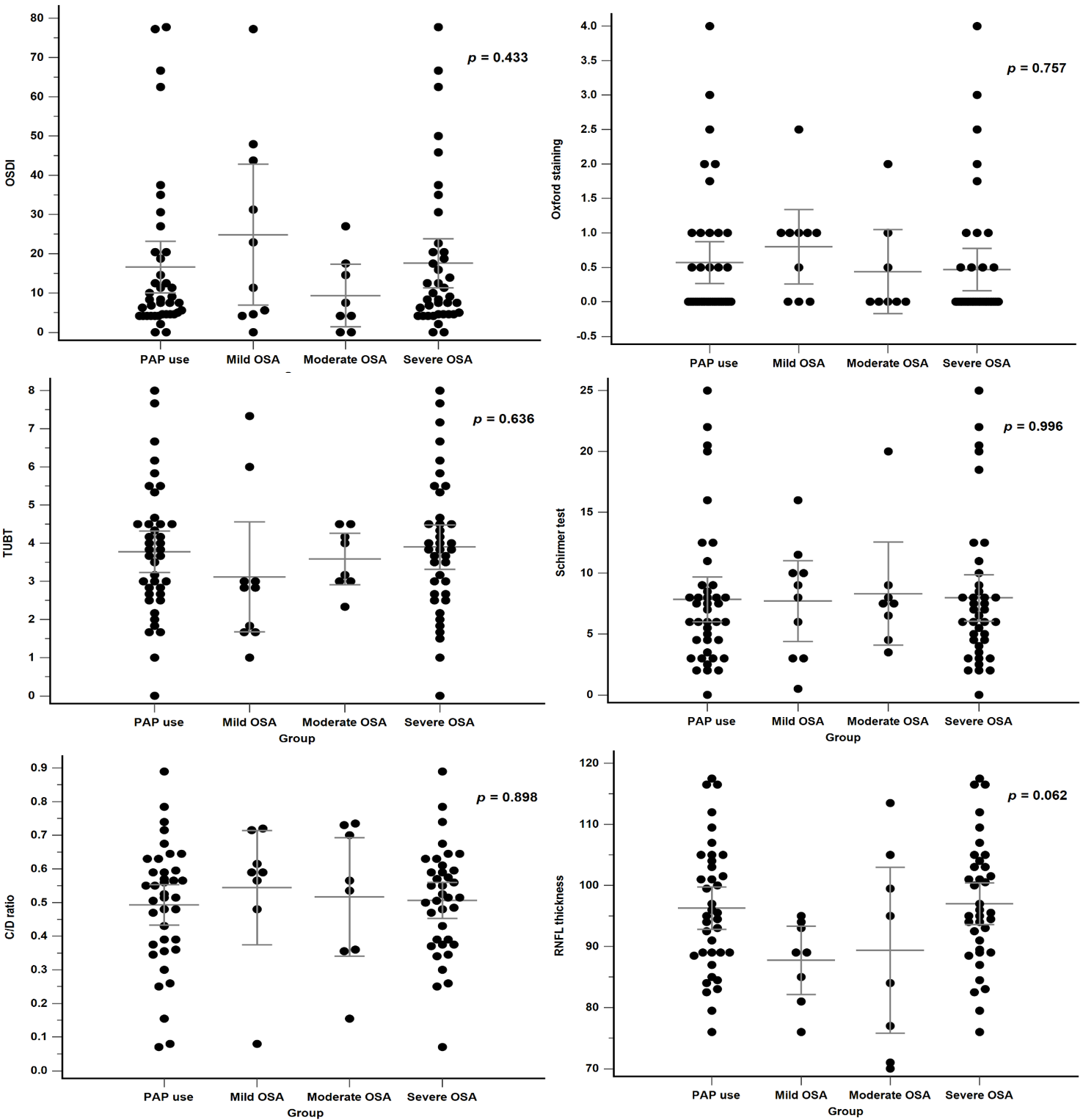

Supplement: Supplementary file 1 [file biomedicines-13-03077-s001.zip › Figure. S2.tif]
